# Can preoperative magnetic resonance imaging be used for sagittal kyphotic flexibility assessment in patients with kyphosis secondary to symptomatic old osteoporotic thoracolumbar fracture?

**DOI:** 10.1186/s13018-023-03624-9

**Published:** 2023-02-23

**Authors:** Kangkang Wang, Feng Zhang, Yunlei Zhai, Wei Zhang, Wen Yin, Lele Sun, Xilong Cui, Haiyang Yu

**Affiliations:** 1 Department of Orthopedics, Affiliated Fuyang People’s Hospital of Anhui Medical University, 501 Sanqing Road, Fuyang, 236000 Anhui China; 2 Spinal Deformity Clinical Medicine and Research Center of Anhui Province, 501 Sanqing Road, Fuyang, 236000 Anhui China; 3 School of Mechatronics Engineering and Automation, Shanghai University, 333 Nanchen Road, Shanghai, 200072 China

**Keywords:** Regional kyphosis angle, Symptomatic old osteoporotic thoracolumbar fracture, Kyphosis, Sagittal kyphotic flexibility, Magnetic resonance imaging

## Abstract

**Objective:**

This study aimed to investigate whether preoperative magnetic resonance imaging (MRI) can be used for sagittal kyphotic (SK) flexibility assessment in patients with kyphosis secondary to symptomatic old osteoporotic thoracolumbar fracture (so-OTLF).

**Methods:**

The authors evaluated the radiographic data of patients with kyphosis secondary to so-OTLF. All patients underwent posterior corrective fusion surgery in the hospital. Spinal sagittal parameters were measured on standing radiographs preoperatively. The regional kyphosis angle (RKA) was also measured on preoperative supine MRI and intraoperative prone radiographs on the surgical frame. The SK flexibility in patients with kyphosis secondary to so-OTLF was defined as the difference from the RKA measured on the standing radiographs to that measured on the intraoperative prone radiographs or preoperative supine MRI. The difference and the correlation between the SK flexibility measured by these two methods were compared and analyzed.

**Results:**

Thirty-seven patients were included. The RKA measured on standing radiographs, supine MRI, and intraoperative prone radiographs were 48.0°, 34.4°, and 32.0°, respectively. Compared with the RKA measured in standing position, the RKA measured on supine MRI decreased by 13.6° (95% confidence interval 11.4°–15.8°), whereas that measured on intraoperative prone radiographs decreased by 16.1° (95% confidence interval 13.7°–18.5°). A linear correlation existed between the SK flexibility measured on supine MRI and that measured on intraoperative prone radiographs, with a mean difference of 2.4° (*R*^2^ = 0.912, *p* < 0.001).

**Conclusion:**

The degree of regional kyphosis deformity was reduced by self-reduction of the intraoperative surgical frame. With a predictive value similar to an intraoperative prone radiograph, preoperative supine MRI can be used for SK flexibility assessment in patients with kyphosis secondary to so-OTLF. The ability to predict the intraoperative degree of regional kyphosis deformity with positioning before an operation may help with surgical planning and patient counseling regarding expectations and risks of surgery.

## Introduction

Given the accelerating aging process of the population, kyphosis secondary to osteoporotic spinal fracture has become a common type of deformity in clinical practice, posing a threat to the physical and mental health of middle-aged and elderly people [1]. Although conservative treatments are effective in temporarily reducing chronic back pain, some individuals with remarkbale sagittal imbalance or neurological deficiencies may still need to undergo surgery [2–5]. Despite the fact that the use of ultrasound bone scalpel, spinal cord monitor, and other medical equipment has improved the safety of surgical treatment, these patients often have complications, i.e., circulatory, respiratory, endocrine, and other systemic diseases. Osteotomy for them is still a great challenge. Only the radiographic parameters of the standing spine full-length radiographs are used to create the surgical plan; this approach carries potential risks, such as significant trauma, increased blood loss, and extended recovery times; some patients may even lose the chance to avail of a surgical cure [6].

In addition to the size of the regional kyphosis angle (RKA) in the standing position and the presence or absence of sagittal imbalance, the surgical plan is also chosen based on the flexibility of the sagittal kyphosis [2, 7]. The kyphotic angle that needs to be corrected during the surgery for flexible kyphotic deformity can be reduced by the self-reduction of the intraoperative position of the surgical frame. However, for rigid kyphosis deformity, the poor mobility of the deformed vertebral body and the adjacent intervertebral space indicate that attempting to restore satisfactory degree correction and sagittal balance without a three-column osteotomy is unrealistic [8–10].

Overextension and overflexion dynamic radiographs are usually used to evaluate the stability of the lumbar intervertebral space. However, the dynamic X-ray film underestimates the reducibility of the kyphosis deformity in these patients. The reason is that the increase in muscle activity and pain of patients during dynamic view shooting prevents some patients from completing movements independently, and these factors are neutralized when patients lie down [11]. We hypothesized that the duration of patient's lying position during preoperative MRI examination was long enough to eliminate such factors as gravity, intractable lumbodorsal pain, and nerve traction pain caused by lumbar spinal stenosis, so as to reduce the compensatory angle that can be reduced and obtain a more realistic kyphosis angle. Although various authors have analyzed the predictive value of supine MRI in predicting TK and LL flexibility for adult spinal deformity, there are few reports about the predictive role of MRI in SK kyphosis flexibility assessment in patients with kyphosis secondary to so-OTLF [12, 13].

The purpose of the present study was to compare the difference and correlation between the RKAs in the supine/prone position and the preoperative standing position, and to determine whether preoperative MRI can be used for SK flexibility assessment in patients with kyphosis secondary to so-OTLF.

## Method

### Patient sample

**Fig. 1:**
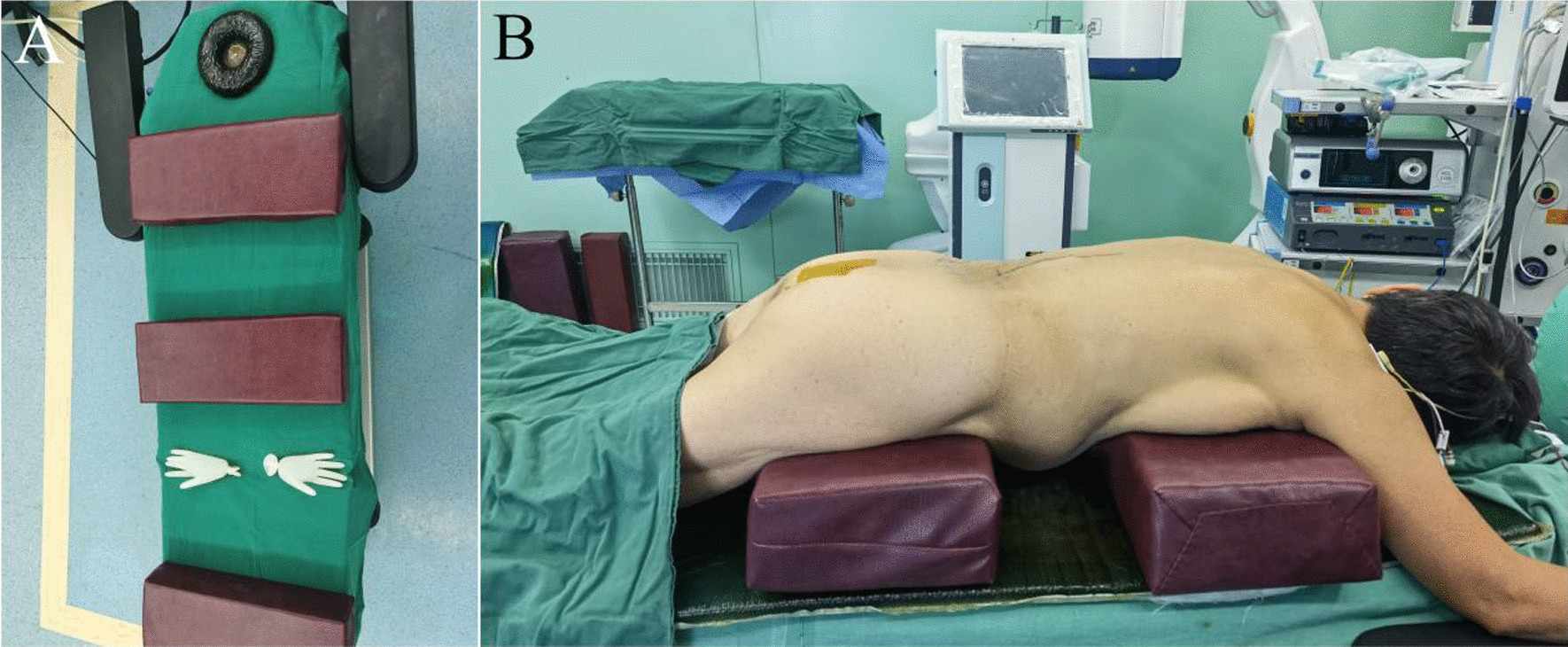
**A** Surgical frame image viewed from the top of the table. **B** Intraoperative prone position. Patients were positioned on the frame with hips and knees in slight flexion. The spinal frame consisted of one anterior chest pad and one pelvic pad

The study group included 37 patients (8 males and 29 females) with kyphosis secondary to so-OTLF. All patients underwent posterior corrective fusion from December 2016 to May 2022. They also underwent preoperative standing X-rays, preoperative supine MRI, and intraoperative prone X-rays. Intraoperative prone RKA was measured using the thoracolumbar radiographs of the spinal frame that were obtained in the prone position after the induction of general anesthesia just before the beginning of the surgery. The surgical frame had one anterior chest pad and one pelvic pad. All patients were positioned on the frame with hips and knees in slight flexion (Fig. 1). The inclusive criteria are as follows: (1) patients who were diagnosed with kyphosis secondary to so-OTLF and have received posterior corrective fusion surgery, (2) fractured vertebrae at T11 to L2 location, (3) RKA measured on standing X-ray radiograph greater than 30°, (4) disease duration longer than 2 months, (5) bone mineral density: *T* score of ≤  − 2.5, and (6) complete imaging data. The exclusion criteria are as follows: (1) spinal tuberculosis, infection, and tumor; (2) incomplete imaging data; (3) the coronal Cobb angle > 10°; and (4) combined with other vertebral fractures and other types of kyphosis, such as degenerative kyphosis.

The Institute’s Institutional Review Committee and Ethics Committee approved the study (Institutional Review Committee No. 2017-01).

### Imaging evaluation

**Fig. 2:**
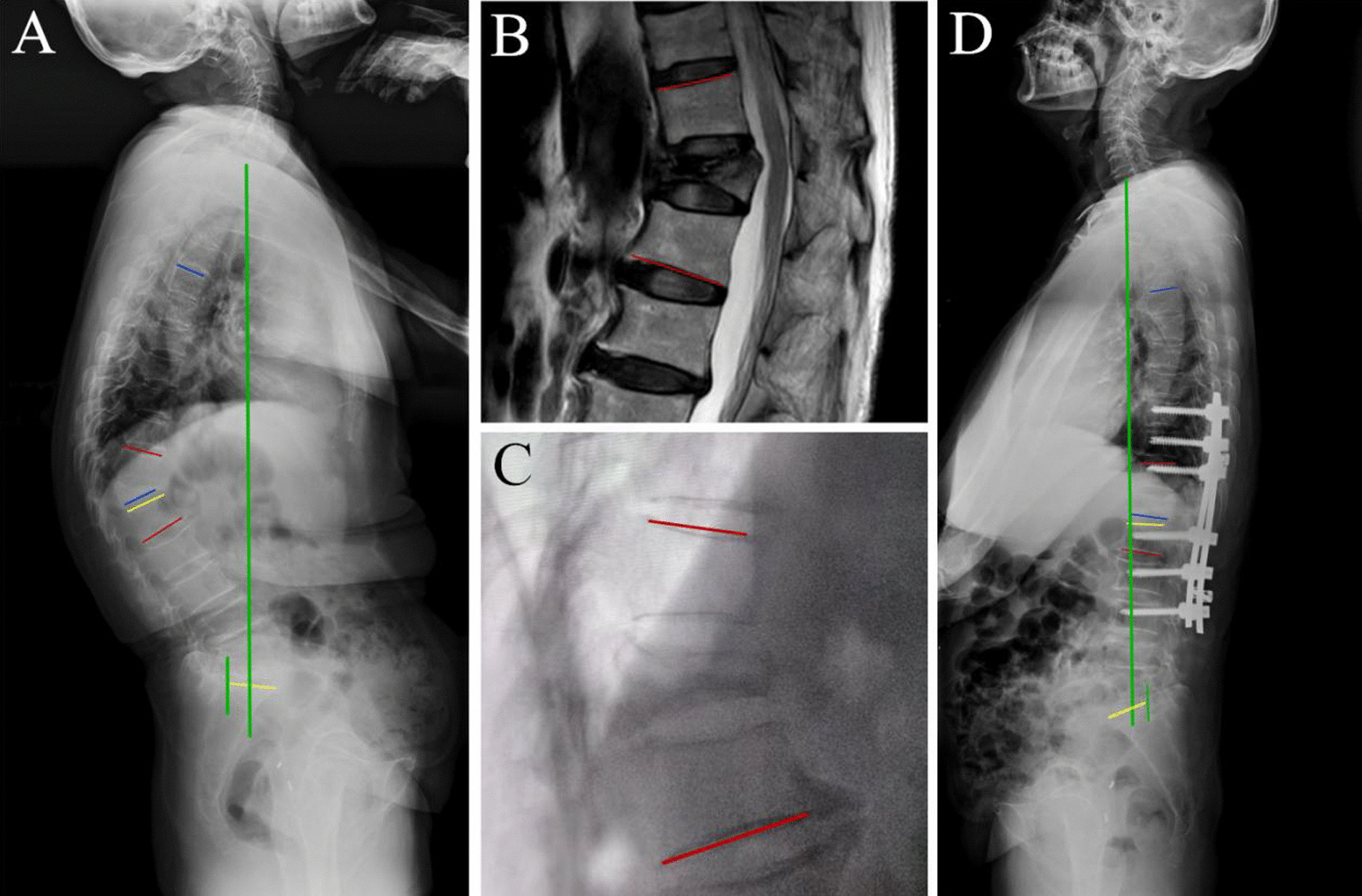
Typical case: female, 68 years old, patient with kyphosis secondary to so-OTLF. She was treated with posterior single-segment Ponte osteotomy and fusion. **A** Image of preoperative standing radiograph; standing RKA: 47.5°, SVA: 26 mm. **B** Image of MRI in supine postion; supine RKA: 27.8°. **C** Image of intraoperative radiograph; intraoperative RKA: 26.7°. **D** Image of postoperative standing radiograph; postoperative standing RKA: 8.1°, SVA: 20 mm

RKA is the angle between the superior end plate of the proximal vertebral body and the inferior end plate of the distal vertebral body of the wedge-shaped vertebra. Thoracic kyphosis (TK) is the angle between the upper end plate of T5 and the lower end plate of T12. Lumbar lordosis (LL) is the angle between the upper end plate of L1 and the upper end plate of S1. Sagittal vertical axis (SVA) is the distance between the center of the C7 vertebral body on the full-length lateral film of the spine to the posterior upper border of S1. If the vertical line is in front of the rear upper edge of S1, then the value is positive; otherwise, the value is negative. A typical case is shown in Fig. 2.

## Result

### General information

Eight men and twenty-nine women met the inclusion criteria. The average age of patients was 66.3 years (53–81 years). The course of the disease was 2–36 months, with an average of 8.2 months. A unilateral vertebral fracture occurred in 25 patients (67.6%). Twelve cases (32.4%) had multiple vertebral fractures (as shown in Table 1).

**Table 1:** Demographics of all the 37 patients

Characteristic	Value
Age (y)	66.3 ± 8.1
Gender	
Men (%)	8 (21.6%)
Women (%)	29 (78.4%)
Fracture location	
T11	2
T12	9
L1	10
L2	4
T12, L1	8
T12, L1, L2	4
Course of disease (month)	8.2 ± 6.7

The average SVA in the standing position before the operation was 51.1 ± 43.1 mm (− 6.2 mm to 19.3 mm). The average RKA was 48.0° ± 12.4° (30.2°–88.8°). The average LL was 30.1° ± 19.5° (− 5.9° to 75.3°). The average TK was 38.2° ± 20.3° (1.4°–79.6°). The imaging parameters of spinal alignment in the standing position are shown in Table 2.

**Table 2:** Spine sagittal radiographic parameters in standing position

Parameter	Preoperative standing radiographs
SVA (mm)	51.1 ± 43.1
RKA (°)	48.0 ± 12.4
LL (°)	30.1 ± 19.5
TK (°)	38.2 ± 20.3

#### Changes in RKA by different positions

**Fig. 3:**
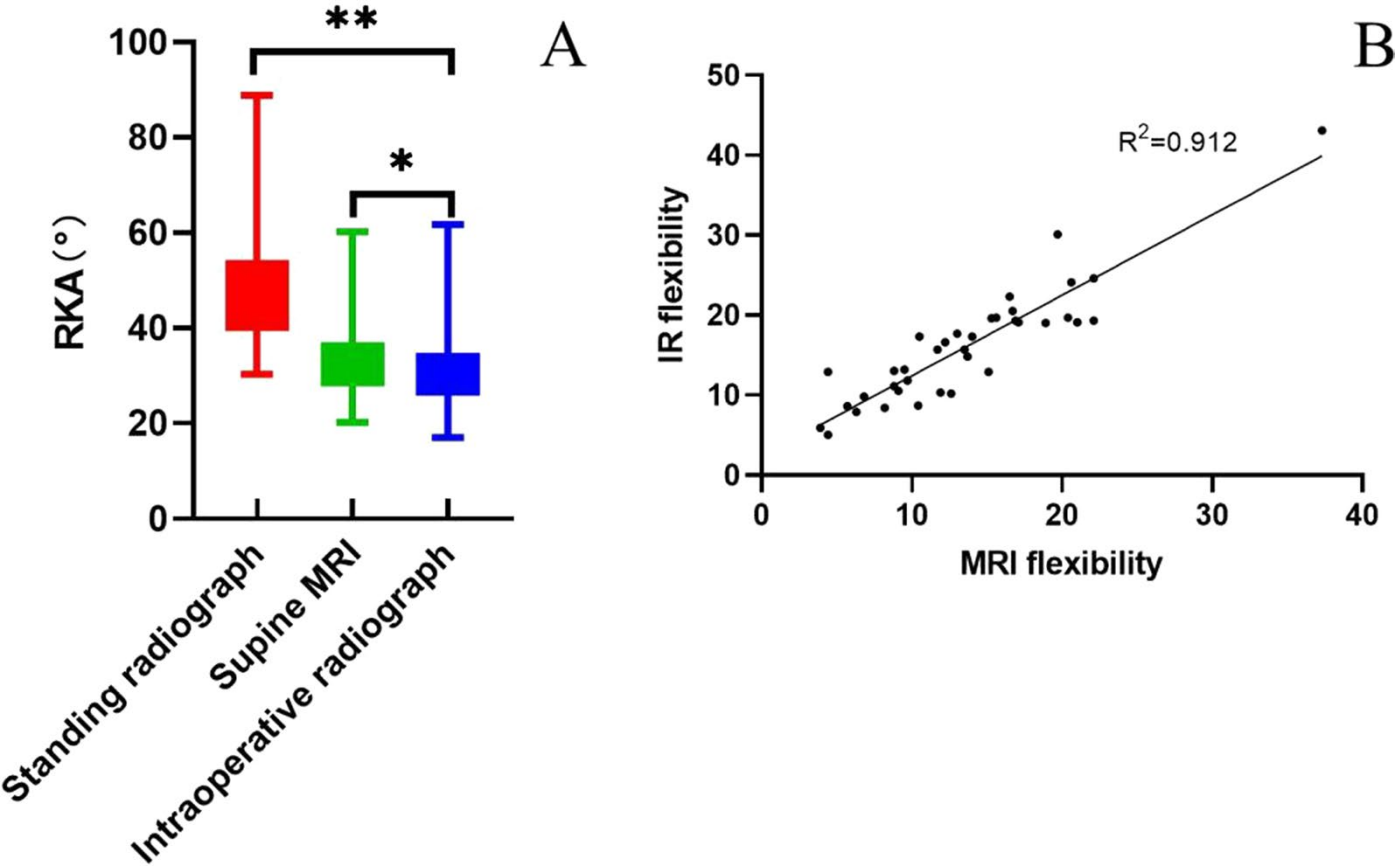
**A** RKAs measured on standing radiographs, supine MRI, and intraoperative prone radiographs were 48.0°, 34.4°, and 32.0°. Compared with the RKA measured in standing position, the RKA measured on supine MRI decreased by 13.6° (95% confidence interval 11.4°–15.8°), whereas the RKA measured on intraoperative prone radiographs decreased by 16.1° (95% confidence interval 13.7°–18.5°). **B** Graph showing the linear dependency between SK flexibility measured on magnetic resonance imaging (MRI) (horizontal axis) and on Intraoperative radiograph (IR) (vertical axis). IR flexibility is on average 2.4° higher than flexibility of sagittal kyphosis curve on MRI (*R*.^2^ = 0.912,* p* < 0.001)

The RKAs measured on standing radiographs, supine MRI, and intraoperative prone radiographs were 48.0°, 34.4°, and 32.0°, respectively (Fig. 3A). Compared with the RKA measured in standing position, the RKA measured on supine MRI decreased by 13.6° (95% confidence interval 11.4°–15.8°), whereas that measured on intraoperative prone radiographs decreased by 16.1° (95% confidence interval 13.7°–18.5°). A linear correlation existed between the SK flexibility measured on supine MRI and that measured on intraoperative prone radiographs, with an average difference of 2.4° (*R*^2^ = 0.912, *p* < 0.001) (Fig. 3B). The mean RKA and SK flexibility are summarized in Table 3.

**Table 3:** Mean RKA and mean SK flexibility measured in different position

Position	Mean RKA	Mean SK flexibility(95% CI)
Standing (°)	48.0 ± 12.4	
Suspine MRI (°)	34.4 ± 10.6	13.6 (95% confidence interval 11.4–15.8)
Intraoperative prone (°)	32.0 ± 9.7	16.1 (95% confidence interval 13.7–18.5)

### Data analysis

SPSS 22.0 (IBM, Armonk, NY, USA) was applied to analyze all data. Continuous variables were expressed as mean ± standard deviation. Counting data Chi-squared inspection was used. The measurement data were evaluated by *t*-test, and *p* < 0.05 means the difference was statistically significant.

## Discussion

Spinal fractures commonly occur in the thoracolumbar segment because it is where the thoracic and lumbar vertebrae meet; this region is unprotected by the ribs and has remarkable mechanical stress and mobility [14]. Inappropriate treatment or even no treatment further aggravates kyphosis; thus, secondary kyphosis deformity is formed, causing intractable low back pain, neurological dysfunction, and other complications in patients and posing a threat to the physical and mental health of middle-aged and elderly people [15–17]. Some studies [18, 19] found that when the RKA is greater than or equal to 30°, patients are at increased risk of chronic pain in the kyphosis area. Pain first occurs at the location of the deformity. Some patients may also complain of pain near the kyphosis area because localized kyphosis alters the normal biomechanics of the patient’s spine, causing premature degeneration of the vertebral levels above and below the deformity. Surgery is usually required when a patient presents with intractable low back pain, remarkable sagittal imbalance, or symptoms of spinal nerve compression. Therefore, a surgical osteotomy is usually performed in the apical region of kyphosis to correct the regional kyphosis of the thoracolumbar segment; thus, the sagittal balance of patients can be obtained and maintained, and the neurological symptoms can be improved [20, 21].

The severity of kyphosis deformity and the presence or absence of sagittal imbalance in the standing position should be considered first when making a surgical plan. The flexibility of the kyphosis deformity must also be evaluated before surgery [1, 11, 15]. RKA, usually measured on a standing spinal lateral X-ray radiograph before surgery, is considered an important indicator of the severity of kyphosis deformity in the spine [22]. Patients are typically in a forward protective posture when standing because of the effects of gravity, persistent back pain, and nerve traction pain caused by spinal stenosis; some elderly patients with weak back muscles cannot even stand entirely, thereby leading to an excessive measured RKA [11, 17]. This measured value does not reflect the actual intraoperative degree of regional kyphosis in patients with kyphosis secondary to so-OTLF, and its clinical application relevance is greatly reduced. Similarly, we found that preoperative standing RKA and intraoperative prone RKA after general anesthesia were not the same in clinical practice. Under general anesthesia, the RKA was reduced to a certain extent; sometimes, it exceeded 50%. Although obtaining full-length X-ray radiographs of a standing spine in the lateral position was straightforward, affordable, and quick, the compensating angle brought on by discomfort and the effects of gravity was not easy to eliminate.

Putto et al. [23] took lateral radiographs with padding at the apex of kyphosis, which can measure the passive reducibility of kyphosis and the extent of the remaining correction (sometimes up to 50%), which may change the entire surgical approach. However, this finding was not investigated in further detail because the authors emphasized only the judgment of intervertebral stability rather than the assessment of flexibility. The X-ray radiographs of lumbar spine flexion and extension positions are often used in clinical diagnosis and treatment of lumbar degenerative diseases to evaluate the stability of the interbody, suggesting that the change in the sagittal curvature of the interbody is the embodiment of flexibility. However, the increase in the patient’s back muscle activity and the aggravation of back pain during the dynamic overextension and overflexion position film limit or prevent the activity of some patients; thus, using dynamic position radiographs to judge sagittal kyphosis flexibility becomes inaccurate [11]. When the patient is lying down during an MRI examination, the patient's back muscles can be completely relaxed, and the protective anteversion brought on by discomfort can also be reduced. Due to the short examination time, these factors cannot be completely eliminated in CT examination [24]. Kaiser et al. [12] found a linear dependency between hyperextension radiograph and MRI flexibility with a mean difference of 9.3° (*R*^2^ = 0.61, *p* < 0.001) by using the imaging data of 18 Scheuermann kyphosis patients. They demonstrated that preoperative MRI has a similar predictive value to bolster-assisted hyperextension lateral radiograph for the Scheuermann kyphosis flexibility assessment. Sharma A et al. analyzed the imaging data from 138 patients with sagittal imbalance. They found that a mean difference of 2.9° exists between the LL measured on supine MRI and that measured on intraoperative X-rays, as opposed to the 5.53° mean difference between standing X-rays and intraoperative X-rays [13]. In patients with flexible deformities (*n* = 24), the lumbar lordosis on MRI measured a discrepancy of 3.08°, as compared to a discrepancy of 11.46° when measured with standing X-ray. This finding showed that preoperative MRI effectively identifies flexible sagittal abnormalities. Moreover, it indicated that the LL recorded on MRI more correctly predicts the intraoperative LL than that measured on standing X-ray in cases of flexible sagittal abnormalities. Similarly, we hypothesized that the MRI examination in supine position could eliminate the factors of protective anteversion caused by intractable back pain, gravity, and nerve traction pain; thus, highly accurate RKA could be obtained.

We evaluated 37 patients (8 males and 29 females) with kyphosis secondary to so-OTLF, with an average age of 66.3 years. The RKAs measured on standing X-ray radiographs, supine MRI, and prone X-ray radiographs before the operation were 48.0°, 34.4°, and 32.0°. Compared with the RKA measured in standing position, the RKA measured on supine MRI decreased by 13.6° (95% confidence interval 11.4°–15.8°), whereas the RKA measured on intraoperative prone radiographs decreased by 16.1° (95% confidence interval 13.7°–18.5°). In the present study, the RKA measured on intraoperative prone radiographs was significantly decreased compared with that measured on standing radiographs (*p* < 0.001). This finding suggests that postural reduction of the surgical frame during surgery significantly reduced the RKA measured on intraoperative prone radiographs. The SK flexibility measured on supine MRI was significantly linearly related to the actual SK flexibility during operation, with an average difference of 2.4° (*R*^2^ = 0.912, *p* < 0.001). This finding indicates that supine MRI can be used to assess SK flexibility in patients with kyphosis secondary to so-OTLF. Moreover, the difference between the RKA measured with preoperative MRI and the actual RKA during the operation was only 2.4°.

Although supine MRI cannot perfectly reflect all the information of the prone position on the operating frame, as a non-weight-bearing imaging examination, it can convey the relevant information about SK flexibility. With a flexible deformity, a large portion of the correction was obtained by positioning only a surgical frame. Moreover, aggressive corrective maneuvers may not be necessary. We used supine MRI to assess the SK flexibility of patients with kyphosis secondary to so-OTLF to develop surgical protocols that can benefit individual patient management. Additionally, preoperative MRI, routinely performed in such patients to exclude neuroaxis pathologies or any compressive cord lesion before, does not increase the economic burden of the patient and the absorption of ionizing radiation.

Given the small sample size, the present study only makes some preliminary discussions on the differences between different positions. It cannot analyze the the apex of the deformity in groups. Thus, a large sample size is required. During the operation, we used the C-arm to perform fluoroscopy with the posterior convex apex as the center. Some images of multiple vertebral fractures need to be spliced, which may have some errors. Furthermore, although different spinal frames might affect RKA in the prone position, we only evaluated one type of frame, and validation using various frames will be required.

## Conclusion

The degree of regional kyphosis deformity was reduced by self-reduction of the intraoperative surgical frame. With a predictive value similar to an intraoperative prone radiograph, preoperative supine MRI can be used for SK flexibility assessment in patients with kyphosis secondary to so-OTLF. The ability to predict the intraoperative degree of regional kyphosis deformity with positioning before the operation helps with surgical planning and patient counseling regarding expectations and risks of surgery.

## Data Availability

The patients’ data were collected in the Affiliated Fuyang People’s Hospital of Anhui Medical University. The datasets used or analyzed during the current study are available from the corresponding author on reasonable request.

## Abbreviations

MRI: Magnetic resonance imaging
SK: Sagittal kyphotic
so-OTLF: Symptomatic old osteoporotic thoracolumbar fracture
RKA: Regional kyphosis angle
TK: Thoracic kyphosis
LL: Lumbar lordosis
SVA: Sagittal vertical axis
IR: Intraoperative radiograph
CI: Confidence interval
